# Supplementary material for: Anti-Inflammatory Activity of Panduratin A against LPS-Induced Microglial Activation
Source: Biomedicines. 2022 Oct 15;10(10):2587. doi: 10.3390/biomedicines10102587 (PMC9599841; doi:10.3390/biomedicines10102587)
Supplement: Supplementary file 1 [file biomedicines-10-02587-s001.zip › biomedicines-1938014-supplementary.pdf]

Supplementary Materials

Anti-Inflammatory Activity of Panduratin A against LPS-Induced Microglial Activation

Sopana Jamornwan, Tanida Chokpanuwat, Kwanchanok Uppakara, Sunhapas Soodvilai and Witchuda Saengsawang

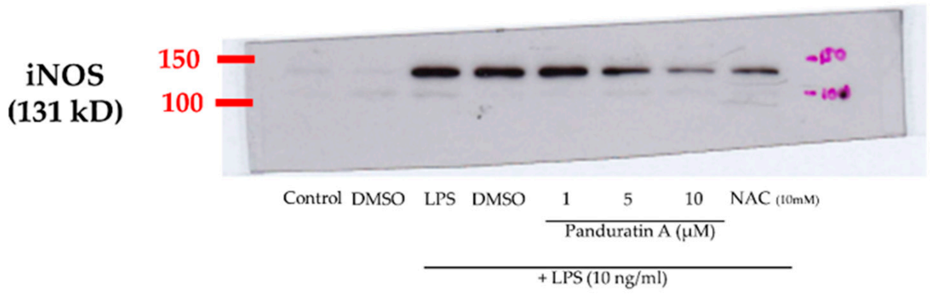

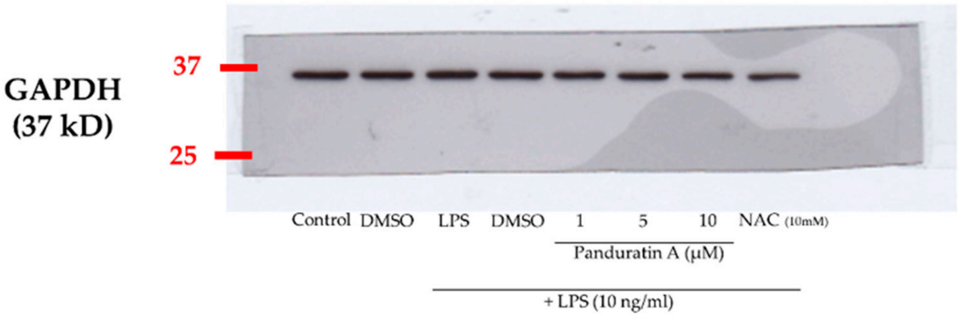

Samples	Densitometry intensity	
	iNOS	GAPDH
Control	142.435	6415.861
DMSO	98.607	6131.154
LPS	7866.518	6141.326
DMSO + LPS	7961.104	6108.205
Panduratin A 1 μM + LPS	7132.569	5901.205
Panduratin A 5 μM + LPS	4650.79	5506.376
Panduratin A 10 μM + LPS	2231.598	4807.962
NAC 10 mM +LPS	2835.305	4336.255

Figure S1. Original images for blots (Figure 2C).

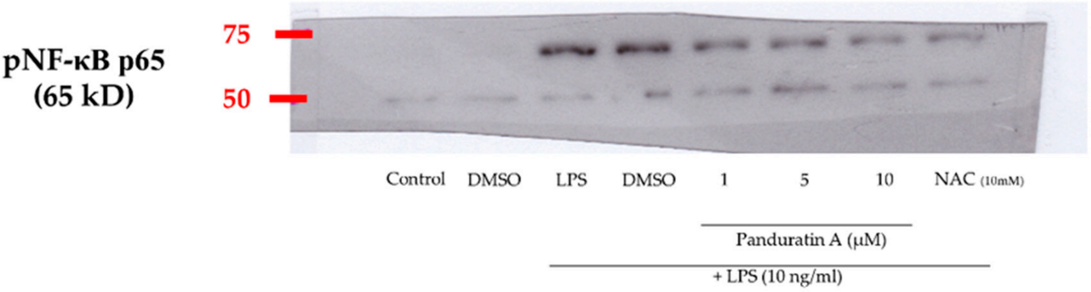

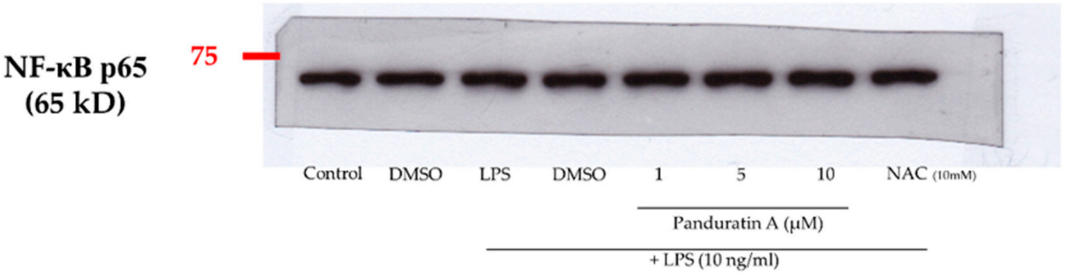

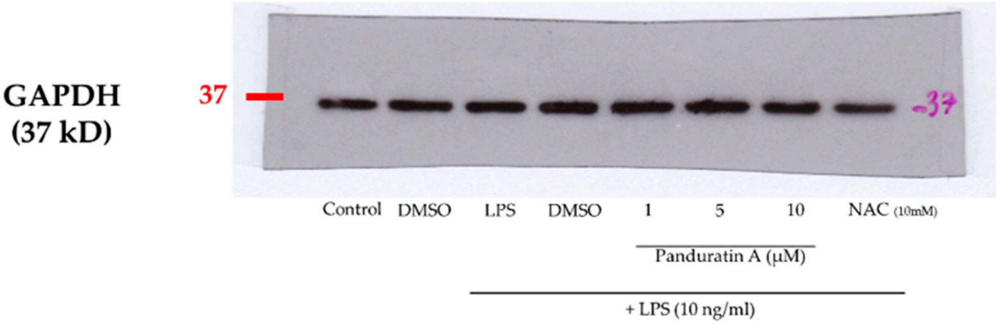

Samples	Densitometry intensity		
	p-NF-κB p65	NF-κB p65	GAPDH
Control	655.648	32894.262	25177.907
DMSO	604.82	35589.798	28143.664
LPS	21754.22	39029.354	26923.179
DMSO + LPS	21088.927	38459.354	29166.007
Panduratin A 1 µM + LPS	15462.572	39088.354	29961.028
Panduratin A 5 µM + LPS	14854.451	39198.869	31702.392
Panduratin A 10 µM + LPS	8978.51	38391.747	25019.25
NAC 10 mM +LPS	7922.61	35491.605	21746.785

Figure S2. Original images for blots (Figure 2D).
